# Polyostotic Fibrous Dysplasia Complicated by Pathological Fracture of Right Femoral Shaft with Nonunion: A Case Report

**DOI:** 10.3389/fsurg.2022.879550

**Published:** 2022-04-12

**Authors:** Qifan Yang, Jing Liu, Lei Tan, Ye Jiang, Dong Zhu

**Affiliations:** ^1^ Department of Orthopedics, The First Hospital of Jilin University, Changchun, China; ^2^ The First Clinical Medical College of Bin Zhou Medical College, Binzhou, China

**Keywords:** fibrous dysplasia, shepherd’s crook deformity, nonunion, osteotomies, femoral shaft fracture

## Abstract

**Introduction:**

Fibrous dysplasia is a benign fibrous bone tumor that accounts for 5% to 10% of benign bone tumors. It can manifest as simple fibrous dysplasia (70%–80%), polyostotic fibrous dysplasia (20%–30%), with approximately the same incidence in men and women. We report a patient with a rare case of multiple fibrous dysplasia combined with proximal femoral shepherd deformity with pathological fracture of the femoral shaft complicated by nonunion. It is necessary to understand the disease in more detail to avoid overtreatment of benign lesions or misdiagnosis of malignant tumors and other diseases.

**Case presentation:**

A 58-year-old man with polyostotic fibrous dysplasia, bilateral proximal femur deformity, Shepherd’s angle deformity, right femoral shaft pathological fracture complicated by nonunion, we under fluoroscopy, in the obvious proximal fracture, take osteotomy, and process the shape of the cut bone fragment to adapt it to the corrected force line, and then restore it back to its original position, using intramedullary nailing technology complete the operation. Three months after the operation, he came to the hospital for re-examination, and an X-ray of the right femur was taken. It was found that the fractured end had a tendency to heal. The patient was instructed to gradually bear weight. After six months of re-examination, the patient could walk with a walker. One year after the operation, the patient could walk without a walker and take care of himself at home. However, there was still stretch-like pain in the right lower back, but it was tolerable.

**Conclusions:**

For patients with polyostotic fibrous dysplasia combined with proximal femoral shepherd deformity and pathological fracture of the femoral shaft with nonunion, osteotomy combined with intramedullary nailing is a simple and convenient way to correct the deformity and obtain correct fracture alignment.

## Introduction

The concept of “fibrous dysplasia” was introduced by Lichtenstein in 1938 (1, 2), which is a benign fibrous bone tumor in which fibrous tissue replaces normal bone tissue with metaplastic young woven bone. It accounts for about 5 to 10 percent of benign bone tumors. It can present as simple fibrous dysplasia (70%–80%), polyostotic fibrous dysplasia (20%–30%), Mccun-Albright syndrome (2%–3%), or Mazabraud syndrome(fibrous dysplasia with intramuscular myxoma) (3). In 1891, von Recklingosen first described that the proximal femur is one of the most common sites. Mechanical stress and repeated fractures result in progressive varus and bowing, typical of shepherd’s curvature (4). Shepherd’s deformity is a deformity that develops through osteoporosis and constant microfracture and fracture repair and is associated with limb pain, lameness, and femoral neck fractures (5).

A classification of proximal femoral deformities has previously been proposed based on the largest cohort of patients with fibrous dysplasia known to us. It is divided into six types according to the neck axis angle and the presence or absence of side axis bending. Types 1 and 2 deformities usually do not progress, while types 3–6 deformities have a higher recurrence rate even with treatment. Type 3 can be corrected with an intertrochanteric osteotomy; types 4 and 5 can be corrected with one or more osteotomies, and type 6 can be fixed with one or more femoral intertrochanteric osteotomies. Osteotomy to correct the deformity. Whenever possible, all associated deformities should be treated with appropriate osteotomy (4) It has also been suggested that polyostotic fibrous dysplasia imaging features at least one of the following: (a) a typical “ground glass” appearance, (b) radiographic Increased or decreased density, (c) bone cyst, or (d) enlarged and/or irregular femoral profile; the presence and extent of the axial cortex and medullary canal (6) consistent with the disease based on the patient’s imaging profile Performance.

Regarding the treatment of fibrous dysplasia, there are many treatment options. Initially, there were simple curettage and bone grafting to treat related deformities. Later, related treatment methods such as osteotomy, plate fixation, and intramedullary nailing appeared (7, 8).

We report a rare case of multiple fibrous dysplasia combined with proximal femoral shepherd deformity in a patient with a pathological fracture of the femoral shaft with nonunion. As far as we know, this is a rare case in China. By providing this case and treatment, we can provide reference experience for medical workers to understand, diagnose and treat this disease in the future.

## Case Report

A 58-year-old man developed bilateral lower extremity deformities when he was young. He developed pain in his right thigh with no obvious cause 8 years ago. He was still able to farm daily and did not receive special treatment. The pain in the right thigh worsened 3 months ago and he was unable to stand immediately. He went to a local hospital and was diagnosed with a fractured right femoral shaft. No surgical treatment was performed, but conservative treatment was given at home. 2 months ago, consciously improved and walked down the ground, fell again, and the pain in the right thigh worsened. Conservative treatment was given at home again. Recently, he was admitted to our hospital because of worsening pain.

**Figure 1:**
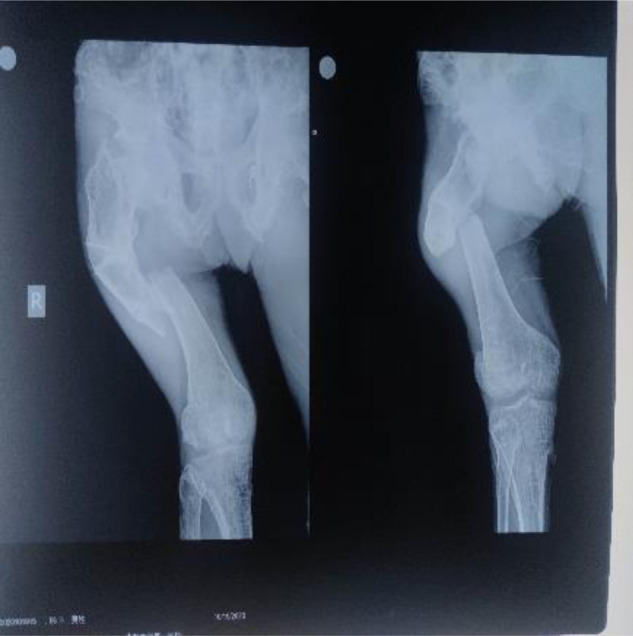
X-ray of the patient on admission.

**Figure 2:**
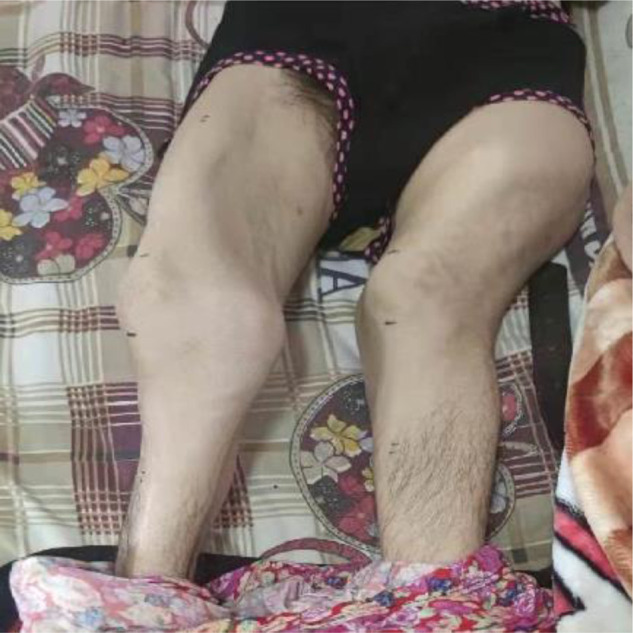
Preoperative external image.

**Figure 3:**
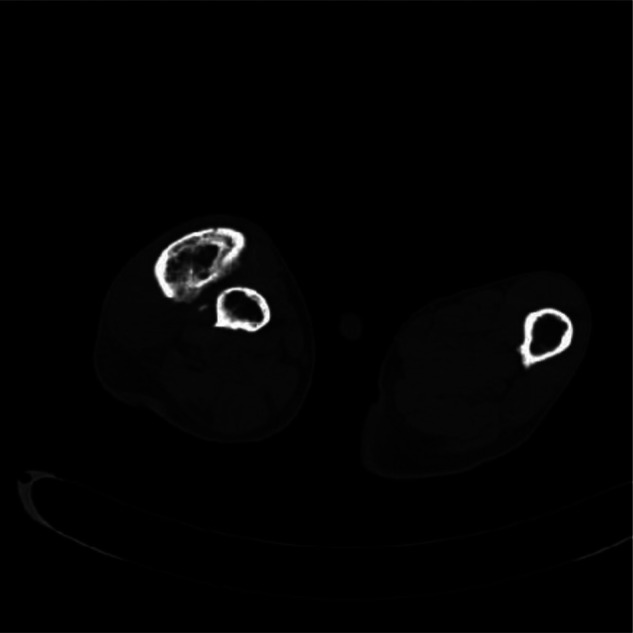
Preoperative CT.

On admission, the clinical presentation was consistent with varus hip deformity, possibly with bilateral femoral neck deformities (Figure 1). In line with Tscherne type I closed fracture soft tissue injury, the remaining manifestations did not have any endocrine disturbance, pigmentation changes, or precocious puberty changes. Both lower extremities were deformed, and both knee joints were huge (Figure 2). In view of the abnormal bone quality of the patient, the imaging examination showed: multiple fibrous dysplasia combined with proximal femoral shepherd deformity, pathological fracture of the femoral shaft complicated by bone Nonunion (Figure 3), multiple fracture lines can be seen on the right femur, old fracture lines and new fracture lines coexist, and the original fractures are malunion. The fractured end of the new fracture was obviously displaced. The patient’s hemoglobin, total number and classification of white blood cells, erythrocyte sedimentation rate, C-reactive protein, calcium, phosphorus, alkaline phosphatase and other blood and serum biochemical indicators and hormone levels were within the normal range. On the second day of hospitalization, the right femoral shaft fracture was treated with open reduction, intramedullary nailing, internal fixation and osteotomy under general anesthesia, and the deformity was corrected and the fracture was fixed in one stage.

**Figure 4:**
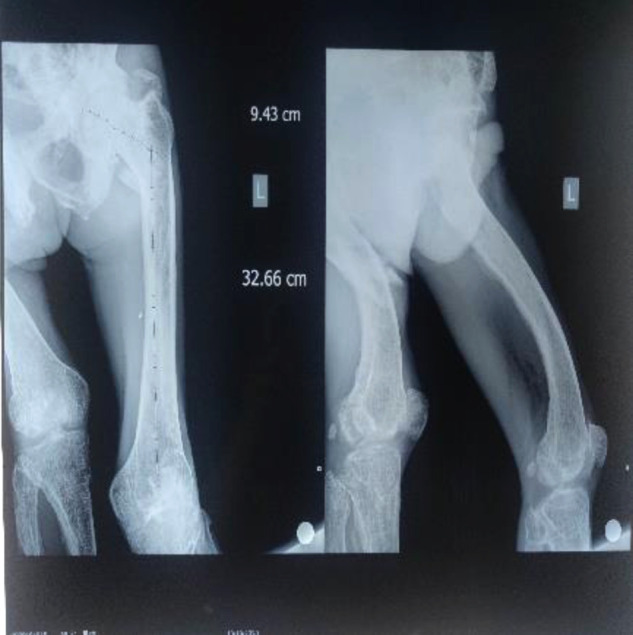
Preoperative imaging of the unaffected femur.

**Figure 5:**
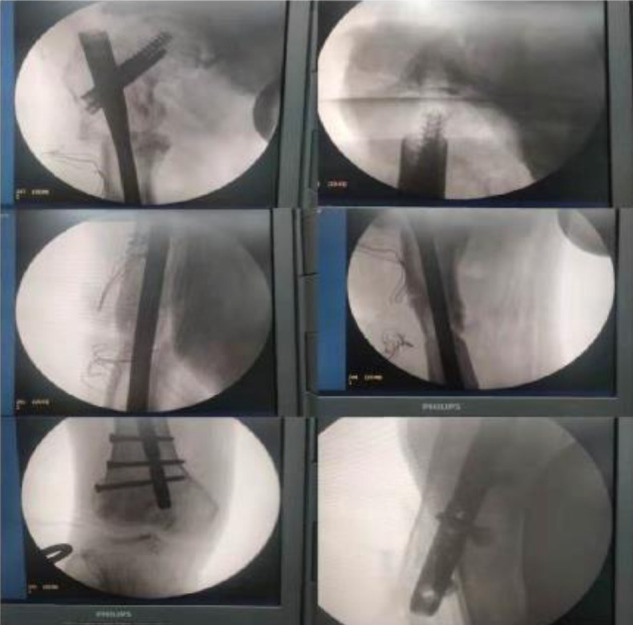
Intraoperative fluoroscopic imaging.

**Figure 6:**
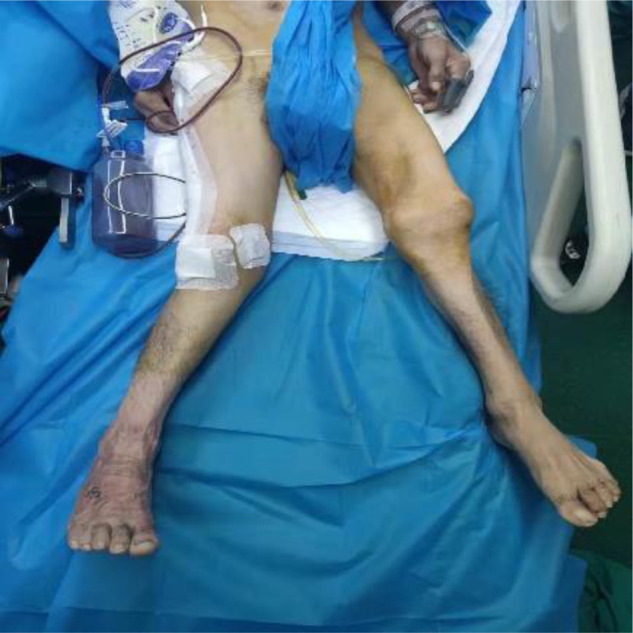
Postoperative external image.

According to the length of the unaffected limb (Figure 4), a special length of intramedullary nail is ordered. This operation is different from the conventional antegrade or retrograde intramedullary nailing. The feature of this case is that after the femoral deformity osteotomy is done, the bone drill is inserted retrogradely from the fracture end to ream the pulp, and the opening is retrograde from the greater trochanter. With the help of the golden finger, the guide pin was introduced retrogradely, and the ball end of the guide pin passed through the orthopedic bone fragment and the distal end of the fracture under direct vision. Postoperative fluoroscopy showed that the distal guide pin was not located in the middle of the medullary cavity, so a 4.0 Steiner wire was used as a block. The intramedullary nail is inserted anterogradely into the medullary cavity, and there is no special treatment for other operations. Because the patient refused to undergo pathological examination, the bone tissue was not taken for examination. This operation successfully completes the fracture fixation and deformity correction through the combination of intramedullary nailing technology and osteotomy. Because the two surgical techniques are mature and simple, the operation time can be greatly reduced and the fracture end can be stabilized. It can reduce the economic pressure of patients, and the central fixation with intramedullary nails has good mechanics, and the blood supply damage to the fracture end is less than that of the steel plate, which can restore the force line of the femur well; however, the femur of such patients is too short, and special reservation The length of intramedullary needles may take a long time and delay the operation date, resulting in complications such as thrombosis, fall off, and fracture pain of the lower extremities, increasing the risk of life and economic burden. The orthopedic bone block has a large resistance during the insertion of the intramedullary needle, so it is necessary to prevent stress fractures and implant fractures (Figure 5). On the third day after the operation, he reported that the right psoas muscle was tense, showing traction-like pain. After physical examination, it was found that the psoas muscle was tense and tenderness was positive. He was given hot compress treatment, and the symptoms were relieved. After the operation, the deformity of the right thigh of the patient was relieved, but the knee valgus was still present (Figure 6).

**Figure 7:**
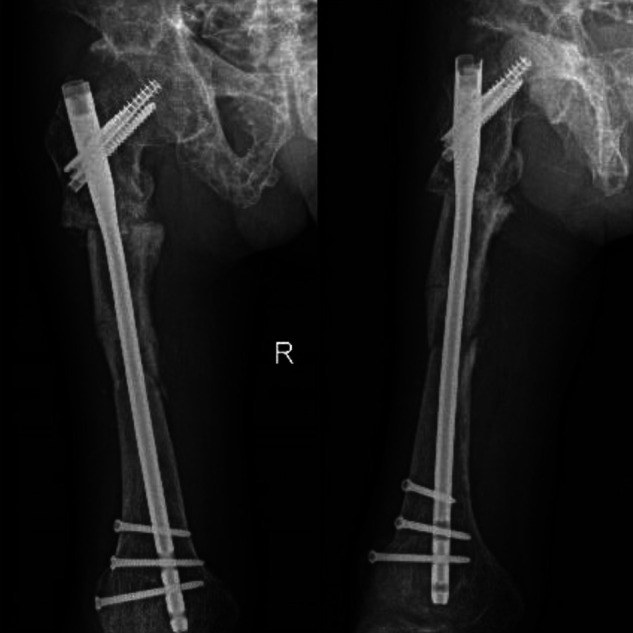
Follow-up 3 months after surgery.

**Figure 8:**
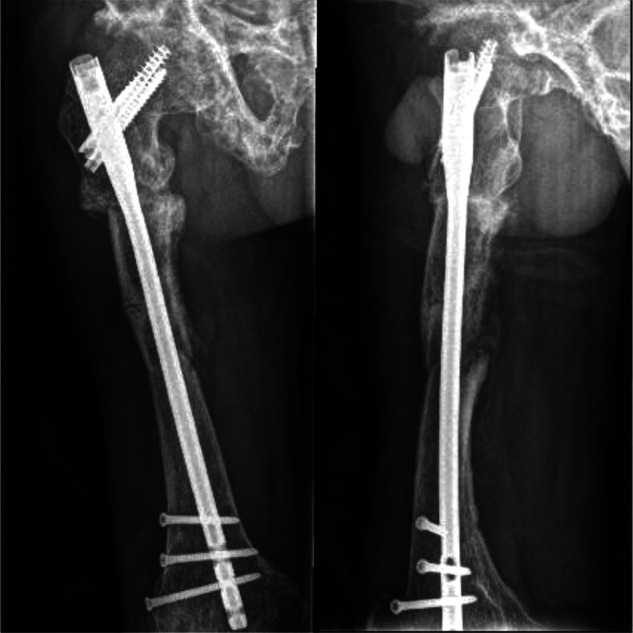
6 months after the operation, the fracture line was further blurred.

**Figure 9:**
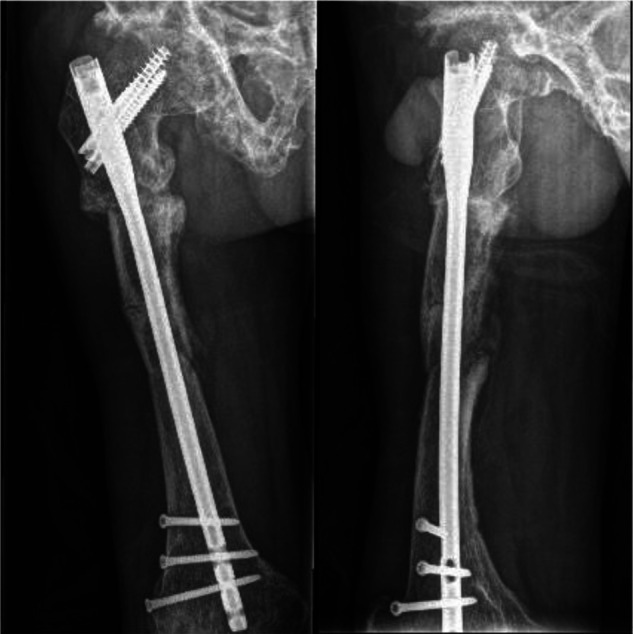
At 14 months postoperatively, the fracture was completely healed.

**Figure 10:**
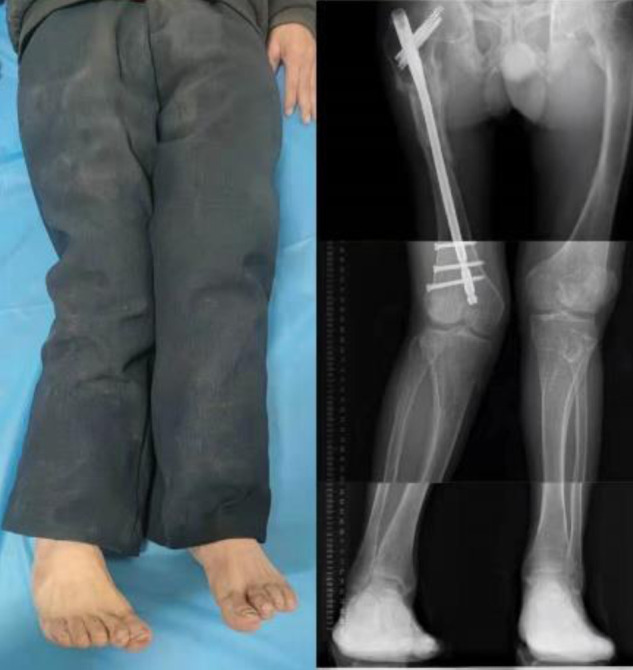
One-year postoperative image.

Three months after the operation, he came to the hospital for re-examination. An X-ray of the right femur was taken. It was seen that the fracture end had a tendency to heal, and the patient was instructed to gradually bear weight (Figure 7); re-examination six months after the operation, the right femur was examined on the front and lateral sides, and the fracture was healed well. Now the patient can walk with the aid of a walker (Figure 8); One year after the operation, the X-ray of the right femur was taken on the front and the lateral side (Figure 9): it was seen that the fractured end had healed. Now the patient can walk without the aid of a walker and can take care of himself at home (Figure 10). However, there was still stretch-like pain in the right lower back, but it was tolerable. This case report has been verbally agreed by the patient and his family (Supplementary Video attached at the end of the article).

## Discussion

The concept of “fibrous dysplasia” was introduced by Lichtenstein in 1938 (2), which is a benign fibrous bone tumor in which fibrous tissue replaces normal bone tissue with metaplastic young woven bone. It accounts for about 5 to 10 percent of benign bone tumors. It can present as simple fibrous dysplasia (70%–80%), polyostotic fibrous dysplasia (20%–30%), Mccun-Albright syndrome (2%–3%), or Mazabraud syndrome (fibrous dysplasia with intramuscular myxoma) (3). Of the affected sites, the proximal femur is one of the most common. As a rare skeletal disease, some scholars believe that its pathogenesis is that the mutation of somatic activation cam regulatory protein Gsa causes normal bone and bone marrow to be replaced by abnormally proliferated osteogenic precursors, resulting in deformity, fracture, dysfunction and pain. The disease can lead to a significant decrease in the patient’s quality of life.

Nonunion is a rare disease in clinical practice. Once it occurs, it can cause huge living and economic burdens to patients and their families. At present, there is no consensus among countries on the definition of nonunion; the US Food and Drug Administration (FDA) defines the disease as: no healing on imaging for 9 months and no significant progress in healing in the past 3 months (9). The Danish Orthopedic Trauma Society defines nonunion as “a fracture that does not heal without further intervention” (10). By consulting relevant information, Protein antibodies, PTH and PTHrP have therapeutic effects on it, but there is no definite clinical trial to prove this conclusion (6); the most widely accepted treatment is still surgery. To strong fixation, restore the stability of the fracture-based.

Regarding the different manifestations and different stages of shepherd’s flexion deformity, it is the most sensible choice to take a targeted treatment method. No matter which customized method is chosen, it is the most important to achieve the correct alignment.

Through reading the literature, the treatment of FD can be divided into conservative and surgical treatment. Conservative treatment includes the use of bisphosphonates to treat the disease, such as zoledronic acid, alendronate, and pamidronate. Long-term results show that it can reduce bone pain (11, 12); Surgical treatment is mostly for children: such as intramedullary nailing + corrective osteotomy, for those with small-sized femurs, the modified humeral intramedullary nail is used, and when the femur grows to a sufficient size, it is replaced with an adult. Femoral intramedullary nailing, there are also valgus osteotomy + lesion curettage + allogeneic bone graft + PHP fixation, and valgus osteotomy combined with growing rods and tension band construct (13–17); for adults, Including dynamic hip screw (DHS) or locking compression plate proximal screw (LCP) combined with intramedullary cortical support allogeneic bone (fibula) fixation, curettage + bone graft + intramedullary nail fixation, some doctors also use “Angled blade plate”, which considers that partially resorbed cortical bone is generally not suitable for locking the proximal screw of the intramedullary nail, and the angled blade plate can be inserted with relative ease, thus providing adequate mechanical support (18–20).

In conclusion, we report a rare case of an adult male with multiple fibrous dysplasia with proximal femoral Shepherd deformity with a pathological fracture of the femoral shaft with nonunion. In this patient’s operation, we successfully reduced the fracture and achieved healing through osteotomy and intramedullary nailing, which effectively improved the patient’s prognosis and saved the patient a lot of treatment costs. This will provide a reference for future clinical diagnosis and treatment of patients with similar conditions.

## Data Availability

The original contributions presented in the study are included in the article/Supplementary Material, further inquiries can be directed to the corresponding author/s.
